# A Concept of Thermographic Method for Non-Destructive Testing of Polymeric Composite Structures Using Self-Heating Effect

**DOI:** 10.3390/s18010074

**Published:** 2017-12-28

**Authors:** Andrzej Katunin

**Affiliations:** Institute of Fundamentals of Machinery Design, Silesian University of Technology, Konarskiego 18A, 44-100 Gliwice, Poland; andrzej.katunin@polsl.pl; Tel.: +48-32-237-1069

**Keywords:** self-heating effect, non-destructive testing, damage identification, vibration-based excitation, vibrothermography

## Abstract

Traditional techniques of active thermography require an external source of energy used for excitation, usually in the form of high power lamps or ultrasonic devices. In this paper, the author presents an alternative approach based on the self-heating effect observable in polymer-based structures during cyclic loading. The presented approach is based on, firstly, determination of bending resonance frequencies of a tested structure, and then, on excitation of a structure with a multi-harmonic signal constructed from the harmonics with frequencies of determined resonances. Following this, heating-up of a tested structure occurs in the location of stress concentration and mechanical energy dissipation due to the viscoelastic response of a structure. By applying multi-harmonic signal, one ensures coverage of the structure by such heated regions. The concept is verified experimentally on artificially damaged composite specimens. The results demonstrate the presented approach and indicate its potential, especially when traditional methods of excitation with an external structure for thermographic inspection cannot be applied.

## 1. Introduction

Due to wide applicability of polymer-matrix composites (PMCs) for manufacturing elements with high load bearing capacity, primarily in aircraft, aerospace, automotive and marine industries, the knowledge about their actual structural condition becomes crucial for their proper operation and estimation of their residual life. Therefore, the non-destructive testing (NDT) of such structures is an important part of their operation. Among the various NDT methods applied to PMCs, including ultrasonic testing, eddy current testing, vibration-based testing, shearography, X-ray computed tomography testing, etc., a group of thermographic methods can be considered as one of the most widespread NDT methods. This is due to several advantages contributing by these methods, such as high speed of testing, and comparatively high sensitivity to particular types of flaws and damage as well as high resolution. In comparison with other NDT methods, infrared thermography (IRT) does not require any couplants and scanning mechanisms, and provides the possibility of detection and identification of both external and internal defects of the material and, during this, does not produce any radiation, unlike, e.g., X-ray-based NDT methods [1].

In NDT studies, the IRT methods are usually limited to the active ones. Following this, an appropriate excitation of a tested structure is necessary, and in classical active IRT this excitation is performed mainly using high-power light sources (e.g., halogen lamps) or lasers. The comprehensive overview of the simplest IRT methods used for NDT purposes, namely pulsed ones, can be found in [2]. To enhance the quality of the thermograms, several other approaches were developed, including lock-in thermography method and its derivatives, such burst phase thermography. However, to eliminate additional external devices, which play the role of a direct heat source, and to control the thermal excitation of tested structures, a subclass of active IRT methods—the vibrothermography (VT)—was developed in the early 1980s [3,4]. This subclass of methods assumes an excitation of a testing structure by vibrations, mainly in the ultrasonic range [5,6,7,8,9,10,11,12], using ultrasonic boosters or actuators integrated with a tested structure, to induce the dissipation processes inside the material in the form of heating. The idea is that heat distribution in a tested structure, resulting from introduced mechanical energy dissipation, is disturbed when the flaw or defect is present, which is detected by the infrared (IR) camera. Among the ultrasonic excitation in VT, other methods were developed, where the approaches are still based on dissipation of the mechanical energy, but the nature of dissipation is different. An interesting approach was proposed in [1], where the authors used electromagnetic induction applied to the reinforcing carbon fiber of a composite to heat the structure locally. In VT methods, the excitation is also possible by subjecting a tested structure to mechanical vibrations with a low-range frequencies. Such an approach was reported in several studies [13,14]. In this case, depending on the materials condition and properties, three mechanisms are possible: energy dissipation due to friction, micro-plasticization, and thermoviscoelasticity [15,16,17]. Note that different energy dissipation mechanisms should be attributed to different materials subjected to testing, e.g., micro-plasticization appears in metallic structures, while thermoviscoelastic effects in polymers and polymer-based composites due to their dominant role in energy dissipation.

The author’s previous studies were focused on describing the fatigue processes in polymeric composites subjected to cyclic loading with appearing self-heating effect, which is a natural consequence of viscoelastic mechanical energy dissipation in such loading conditions. During experimental studies performed for resonant vibrations on composite elements [18,19], the characteristic temperature distributions, caused due to the appearance of the self-heating effect, were observed. These distributions coincide with modal shapes of vibration, when the tested structures were loaded on their natural frequencies of vibration. The self-heating effect in polymeric structures was used for thermal excitation in several studies (see e.g., [13,20,21]), however, in the first case, the self-heating effect was just a tool for observation of an initiated crack, while, in the two latter cases, the excitation in the ultrasonic frequency range was performed.

Following the results of performed experimental studies [19], it is clearly observable that the regions of temperature increase are related with the highest values of deflection in particular modal shapes; thus, there is almost no heating in the nodes of the modal shapes. However, to have a possibility of identification of a damage in all possible locations, it is necessary to have a full coverage of a tested structure. Therefore, it is essential to consider several modal shapes during an excitation of a tested structure.

The aim of this study is to present a novel approach of the self-heating-based VT method dedicated for PMCs, where the excitation is performed on several natural frequencies simultaneously to excite the whole surface of a tested structure. Based on the results of experimental studies performed on artificially damaged composite specimens, it was shown that the proposed approach can be an effective alternative for NDT of polymeric composite structures, where other IRT NDT methods cannot be applied.

## 2. Materials and Experiments

### 2.1. Specimens Configuration

The specimens used for tests were manufactured from 14-layered epoxy-based composites reinforced by unidirectional E-glass fabric purchased from Izo-Erg S.A. (Gliwice, Poland). The dimensions of specimens were as follows: width of 10 mm, thickness of 2.5 mm, and length of 250 mm (note that the effective length, i.e., the length of the part of a specimen not clamped in the holders, was of 200 mm). To analyze the effectiveness of the proposed approach in damage identification, 9 equidistant notches with a depth of ca. 0.5 mm and distances between them of 25 mm were introduced on the specimens’ top surface. Additionally, to ensure appropriate thermal emissivity, the specimens were covered with a silicone black matt heat-resisting enamel.

### 2.2. Experimental Setup and Testing Procedure

**Figure 1:**
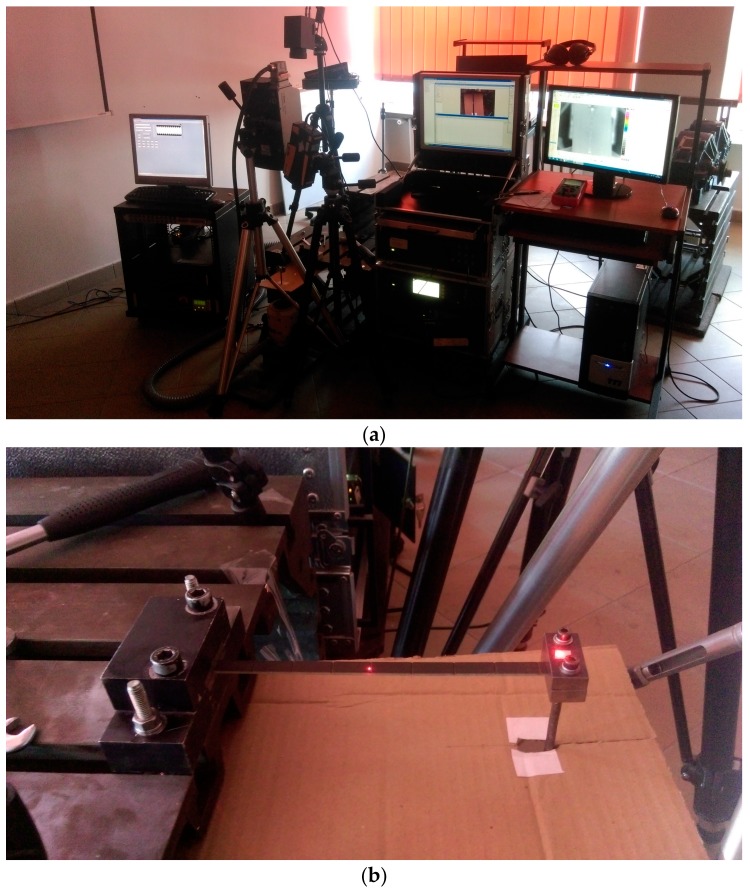
Experimental setup: (**a**) general view; (**b**) detailed view on the specimen.

The experimental setup consisted of a holder mounted to the steel fixed block, in which a tested specimen was clamped, and the second holder clamping the specimen on the opposite side, but also connected through the steel stinger with an electrodynamic shaker used for excitation of mechanical vibrations in this specimen. The shaker was connected through the power amplifier to a PC, where the excitation signal was generated from the application. The infrared camera as well as heads of two laser Doppler vibrometers (LDVs) were placed perpendicular to the specimen’s top surface. The view of the whole experimental setup and a closer view of the tested specimen are presented in Figure 1a,b, respectively.

**Figure 2:**
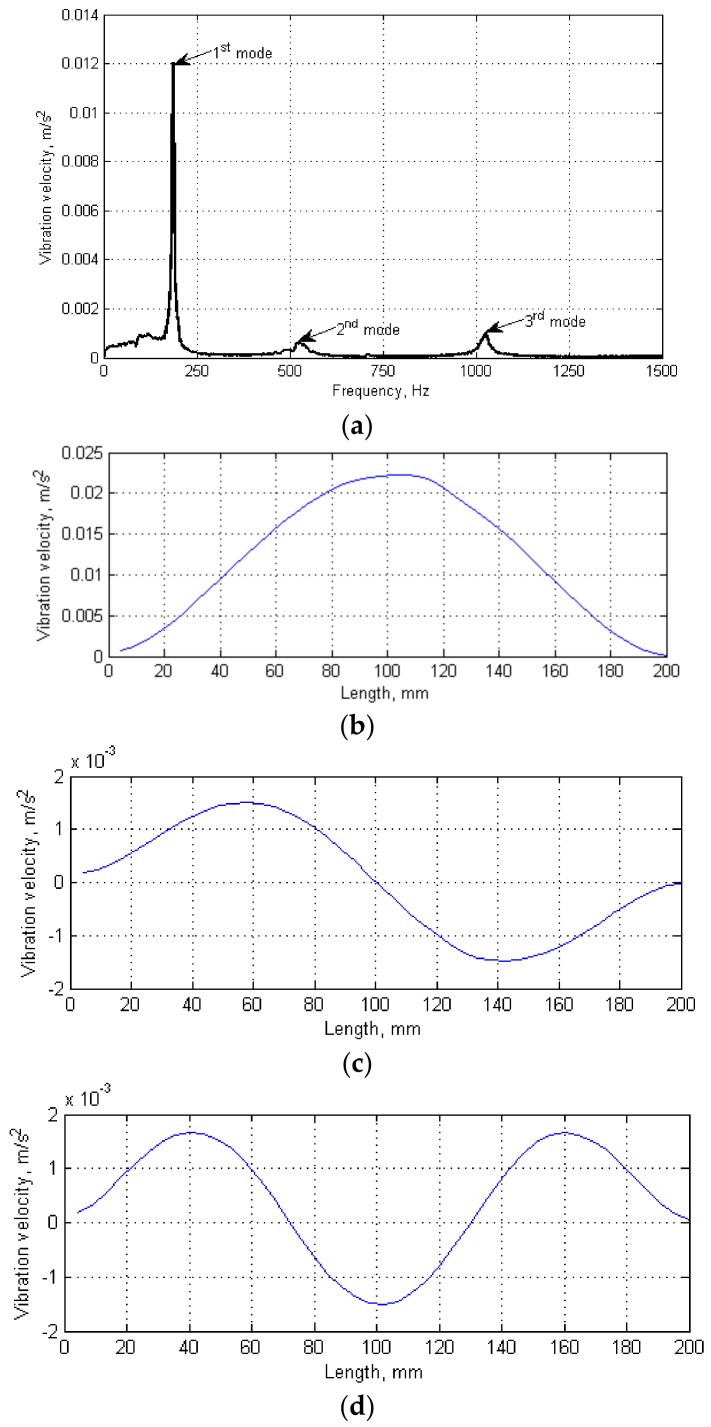
Results of the modal analysis: (**a**) frequency response function; and (**b**–**d**) modal shapes corresponding to selected resonances.

The testing procedure consisted of two steps. After clamping a tested specimen in holders and sticking the reflective tape on its surface to ensure appropriate reflectivity of a laser beam of a scanning LDV, the modal analysis was performed. The second LDV was oriented and focused on the upper surface of the smaller holder connected with a shaker to acquire the reference signal. The modal analysis was performed in the frequency range of 0–1500 Hz with a frequency resolution of 1.5625 Hz by excitation a tested specimen with a pseudo-random signal to acquire the wide-band frequency response. The number of scanning points during the modal analysis was set to 45 with 5 averaging cycles to ensure high accuracy of estimated natural frequencies. As a result of the modal analysis, the frequency response function (FRF) was obtained (Figure 2a). From FRF, the resonance peaks were selected, and the corresponding natural frequencies and modal shapes (Figure 2b–d) were acquired.

During the second step, VT testing was performed using the determined natural frequencies of vibration (185.94, 518.75, and 1025 Hz, respectively). To evaluate the effectiveness of damage detection and identification in the tested specimens, the excitation was performed following various loading scenarios: Firstly, the loading was performed with single harmonic signals with frequency values corresponding to the determined natural frequencies for tested specimens. Second, combinations of these harmonics (i.e., second and third one, and all three harmonics) were used in the form of a multi-harmonic signal. The loading was performed using the own-developed software for generation of this multi-harmonic signal. Note that, in all above cases, the magnitudes of particular harmonics in multi-harmonic excitation signals were the same. To preserve the same conditions as during the modal analysis (to excite vibrations exactly with the determined natural frequencies), the loading was performed using the same electrodynamic shaker without any interference to holders and clamping conditions. The thermal response was registered by the IR camera focused on the tested specimen with a sample rate of 2 thermograms per second. The duration of the test was limited to two minutes, when the stabilization of the self-heating temperature distribution was observed. To separate thermal responses of a tested specimen and an electrodynamic shaker, a cardboard thermal screen was used. This allows for eliminating a thermal signature of the electrodynamic shaker from acquired thermograms, since the shaker generates heat during intensive vibrations, which may disturb the observed temperature distributions on a tested specimen.

## 3. Results and Discussion

**Figure 3:**
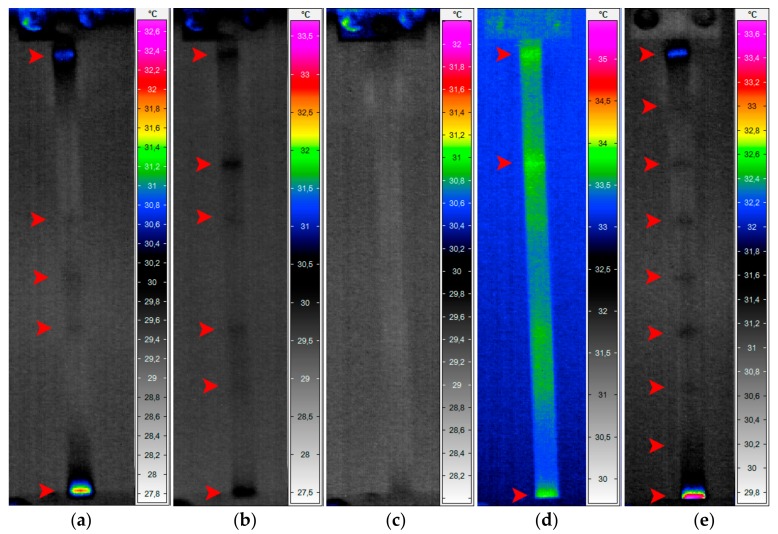
Resulting temperature distributions during loading on: (**a**) first harmonic; (**b**) second harmonic; (**c**) third harmonic; (**d**) second and third harmonics; and (**e**) first, second, and third harmonics.

During the first step of experimental studies, the tested specimens were loaded according to the above described loading programs, namely, using harmonic signals corresponding with natural frequencies of vibration. The second step consisted of studies with loading using multi-harmonic signals corresponding with multiple natural frequencies, namely second and third harmonics, and all three harmonics. The resulting temperature distributions for these cases are presented in Figure 3.

The observed results show a strong dependence between the excited modal shape and detectability of damage. In the case when the tested specimen was excited by the harmonic signal with its first natural frequency (Figure 3a), the temperature increase coincides with the maximal magnitudes of vibration on this mode (cf. Figure 2b) and is also observed in the regions of stress concentrations, i.e., near the clamps in the investigated case. Following this, only five of nine existing damage locations were identifiable: near the clamps and in the central part of the tested specimen. Similarly, in the second case (Figure 3b), when the tested specimen was loaded with its second natural frequency, the damage locations were observable in the regions of the highest vibration magnitudes for this modal shape (cf. Figure 2c), and six of nine damage locations were identified. The loading of the tested specimen with the third natural frequency did not give expected temperature distribution and a possibility of localization of damage, since the magnitude of vibration with this frequency was not high enough to excite the characteristic temperature distribution; therefore, none of thermal responses in the location of notches were observed in this case (Figure 3c). This observation leads to the conclusion that the excitation of tested structures on higher natural frequencies does not always give a possibility of damage identification.

To overcome the limitation of the observed patterns, i.e., the heating-up of the tested structures in particular regions only, and therefore, hiding several damage locations, it was decided to construct multi-harmonic signals composed of harmonics with natural frequencies. In the case, when the specimen was excited using a multi-harmonic signal of second and third natural frequencies (Figure 3d), only three of nine damage locations can be clearly identified. In this case, due to covering of the second and third modal shapes (see Figure 2c,d), the effect of compensation occurred, which resulted in blurring the regions where the damage locations were well identifiable for the case of excitation with second natural frequency only (cf. Figure 3b). Finally, when the tested specimen was loaded with a multi-harmonic signal composed of all three determined natural frequencies, all nine damage locations were identifiable. This allows concluding that the proposed approach is effective when the excitation signal is composed in such a way that the heating regions attributed to particular modal shapes (and corresponding natural frequencies) cover the entire surface of a tested specimen.

Since a series of thermograms was acquired for every tested case, the analysis of influence of the excitation duration was possible. Following the performed observations, it can be stated that the best results (in terms of temperature selectivity and sharpness of thermograms) were obtained at the beginning of temperature stabilization after exciting the self-heating effect in the tested specimen. The duration of excitation of a specimen depends on the magnitude of applied vibrations, but usually it is enough to excite a tested structure up to 100 s to obtain appropriate thermal response, which makes it possible to identify the damage. This leads to an analogy of the proposed approach to the transient thermography method, since, in the latter method, the thermal stimulation is performed for a longer time, which makes it possible to improve detectability with respect to classical IRT methods, such as pulse thermography.

## 4. Conclusions

This paper presents a proof of concept of a new vibrothermographic approach for non-destructive testing of polymeric composite structures by means of inducing in a tested structure the self-heating effect. This approach allows resigning from using external heat sources (traditional in active thermography methods) by their substitution with mechanical energy dissipation initiated from the excitation of mechanical vibrations in tested structures. The performed experimental studies clearly indicated that the excitation of specimens with harmonic signals corresponding to their natural frequencies of vibration, including the higher natural frequencies, does not allows for the thermal excitation of the entire surface of a tested specimen. The proposed approach based on multi-harmonic excitation signal composed from the first three natural frequencies of the tested specimen allows identifying all existing artificial damage locations, which confirmed experimentally the initial idea of this method.

Since the obtained results in the proof of concept studies are not clear enough, additional studies on its improvement are planned. In particular, other combinations of excitation using multi-harmonic signals will be tested. The newest results obtained to-date on sensitivity of the proposed methods show that the notches of 0.25 mm in depth (10% of total thickness) in the case of observation of the tested specimen from the opposite side to the damaged surface are still identifiable. However, the sensitivity may be increased by application of advanced signal and image processing methods, which are planned to be applied in further studies.

The proposed method may find practical application in such cases when the excitation by external heat sources or excitation with ultrasonic boosters is impossible or impractical. These cases are concerned with a lack of direct access or limited access to the structure, when the thermal excitation can be provided by global vibration excitation only, e.g., interior composite aircraft load-bearing elements. Moreover, the proposed method needs no baseline measurements, i.e., the damage can be detected and identified in a direct way on a tested structure, not by comparison with a healthy structure. The method provides quick measurement and the diagnostic result is simple for interpretation, which makes the decision-making process easy. An additional advantage of the proposed method with respect to other thermographic approaches is the possibility of covering large surfaces, which makes the proposed approach much faster. This advantage is connected to another one: having various modal shapes, the end-user can control the thermal excitation process by selection of such modal shapes, which allows heating in the expected region, or selecting several modal shapes, as presented in the experimental study, to cover the entire surface of a tested object. This solves the problem with non-uniform excitation when flash lamps are used in the pulse thermography approach as well as the problem of non-uniform heating of tested structures with complex geometry. Following this, the proposed approach can be considered as a profitable alternative NDT for structural elements made of polymeric composite materials.

## 5. Patents

The obtained promising results allow submitting a patent application to secure the rights to the developed method [22].
